# Post-traumatic stress disorder among heart disease patients: a clinical follow-up of individuals with myocardial infarction in the Tromsø Study

**DOI:** 10.1186/s12888-023-05431-2

**Published:** 2023-12-12

**Authors:** Geir Fagerjord Lorem, Eva T. Næss, Maja-Lisa Løchen, Kjersti Lillevoll, Else-Marie Molund, Assami Rösner, Sigmund Lindkvist, Henrik Schirmer

**Affiliations:** 1 Department of Psychology, Faculty of Health Sciences, UiT The Arctic University of Norway, Tromsø, Norway; 2 Department of Internal Medicine, University Hospital of North Norway, Tromsø, Norway; 3 Department of Cardiology, University Hospital of North Norway, Tromsø, Norway; 4 Department of Cardiology, Akershus University Hospital, Nordbyhagen, Norway; 5 Department of Clinical Medicine, Campus Ahus, University of Oslo, Oslo, Norway; 6 Department of Medicine, Finnmark Hospital Trust, Hammerfest, Norway; 7 Department of Clinical Medicine, UiT The Arctic University of Norway, Tromsø, Norway

**Keywords:** Post-traumatic stress disorder (PTSD), Myocardial infarction, Mental health, Mental trauma, Stressful life events, The Tromsø Study, Public health, Comorbid mental health

## Abstract

**Background:**

Myocardial infarction is likely to be experienced as a life-threatening and potentially traumatic event. Approximately one-third of patients with myocardial infarction experience clinically significant symptoms of anxiety/depression. However, it is unclear how many of these patients experience these symptoms because of post-traumatic stress disorder (PTSD). We conducted a clinical screening of individuals with a confirmed myocardial infarction diagnosis. Our goal was to examine the prevalence of PTSD in myocardial infarction patients and study how PTSD symptoms were associated with exposure to potentially traumatic events.

**Method:**

This is epidemiological research with a cross-sectional design following up participants from the Tromsø Study with a confirmed diagnosis of myocardial infarction. We sent invitations to participants in the Tromsø Study with clinically significant self-reported anxiety or depression symptoms following myocardial infarction. A cross-sectional sample of *N* = 79 participants (61 men and 18 women) was collected. During an interview, participants completed the Stressful Life Events Screening Questionnaire and the PTSD checklist PCL-5.

**Results:**

We found nine participants (11.6%) with probable PTSD. This was significantly higher than the postulated population prevalence in Norway (*p* < 0.015). We found no direct association between myocardial infarction as illness trauma and symptom levels (*p* = 0.123). However, we found a significant linear trend (*p* = 0.002), indicating that symptom severity increased proportionately as the number of post-traumatic events increased.

**Conclusion:**

PTSD prevalence in myocardial infarction patients was related to lifetime exposure to traumatic events, not the myocardial infarction event alone. More research is required to examine the interaction between myocardial infarction and PTSD. Clinicians should be aware that anxiety or depression symptoms after MI could be secondary symptoms of PTSD.

**Supplementary Information:**

The online version contains supplementary material available at 10.1186/s12888-023-05431-2.

## Introduction

It is widely recognized that heart disease is related to symptoms of anxiety or depression [1]. Post-diagnostic comorbid mental health issues among heart disease patients are well described [2–11], but the main research focus has been on mood disorders (i.e., anxiety/depression). We found in a previous study that 32.9% of patients with myocardial infarction (MI) reported clinically significant symptoms of anxiety and depression [12]. The prevalence is higher than in the general population [13]. However, it is important to note that experiencing both anxiety and depressive symptoms does not automatically mean that an individual is suffering from a mood disorder. These symptoms can also be secondary to trauma and stress-related disorders, such as post-traumatic stress disorder (PTSD).

PTSD is unique among mental disorders because it has a bidirectional relationship with cardiovascular disease (CVD). On the one hand, exposure to traumatic events and subsequent development of PTSD can harm physical health and increase the risk of early CVD, and in turn, worsen the prognosis of the CVD [14–16]. PTSD is a condition that causes elevated and dysregulated long-term stress response, leading to an increase in blood pressure and heart rate. This can increase the likelihood of developing coronary heart disease [17, 18]. PTSD can have biological consequences such as hypothalamic–pituitary–adrenal axis dysregulation, dysfunction of the autonomic nervous system, and chronic inflammation. These effects can increase the risk of developing new cardiac events or negatively impact the prognosis [19, 20].

A person may develop post-traumatic stress disorder (PTSD) after experiencing one or multiple traumatic events, including but not limited to war, physical assault, sexual violence, kidnapping, hostage-taking, terrorist attacks, torture, natural or human-made disasters, and severe accidents. Life-threatening illnesses are not necessarily considered traumatic events, and medical incidents that do qualify as traumatic events are typically sudden, catastrophic events [21]. MI is likely to be experienced as a life-threatening situation and might become a traumatic event [22]. MI patients may experience severe stress, leading to the onset of PTSD symptoms in vulnerable individuals [23]. In addition to the subjective perception and reaction to the cardiac event (e.g., believing one will die), other risk factors are equally important, such as previous traumatization, previous heart disease, negative affect (in hospital), lack of social support, and depression. Intense fear may also be increased by external frightening elements during hospital treatment, especially when the demand for emergency services exceeds the ability of physicians and nurses to provide quality care within a reasonable time [19].

The lifetime occurrence of PTSD in Norway is 2.5%, while in the USA, it is approximately 8% [24–26]. According to a Norwegian study, 22% of MI patients met the criteria for a diagnosis of PTSD [27]. A Swedish study found a 21% prevalence of PTSD after surviving an out-of-hospital *cardiac arrest* [28]. Other studies show an average of 12% (range 0%-38%) prevalence of PTSD [29].

We wanted to identify *PTSD among* patients with myocardial infarction and examine their experience of potentially traumatic events (PTEs). The Tromsø Study is a prospective cohort study in Norway. We invited all participants registered with a confirmed MI diagnosis and significant self-reported mental health symptoms. All underwent screening to identify individuals with a plausible PTSD diagnosis. The objective was to gather evidence of probable PTSD and examine how symptom severity levels are associated with disease and other PTEs.

## Method

### Sample and design

This is a cross-sectional epidemiological study that followed up participants from the Tromsø Study who were diagnosed with MI. The Tromsø Study is a prospective cohort study consisting of seven repeated population health surveys. They are referred to as Tromsø1-7 and were carried out in 1974, 1979–80, 1986–87, 1994–95, 2001, 2007–08, and 2015–16. The study includes large, representative samples of the Tromsø population, with the invitation of whole birth cohorts and random samples [30]. The Tromsø Study collects data on incident cases of MI by linkage to the discharge diagnosis registry with a search for relevant diagnoses at the University Hospital of North Norway (including outpatients) for the participants using the unique national identification number. The systematic registration of MI events started in 1974 and ended in 2014. The incident cases of MI were identified retrospectively and determined by an endpoint committee using medical records and notes. This process is described in detail elsewhere [31].

We used two samples recruited from the Tromsø Study participants (Tromsø7) and the Department of Cardiac Medicine of the University Hospital of North Norway (UNN). The inclusion criteria were men and women with a confirmed MI diagnosis. From a total of 818 participants with a validated MI diagnosis among the Tromsø7 participants, we identified 208 individuals with both MI and significant symptoms of anxiety or depression (Hopkins Symptom Checklist score > 1.65). Those still alive (*N* = 140) were contacted by the Tromsø Study with a written invitation to participate in a follow-up interview. Sixty-two participants (response rate 44.3%) consented and participated in a clinical screening.

We invited men and women of varying ages and types of infarctions (STEMI/NSTEMI) from UNN. We included former patients with at least one year since the MI event. A partner at UNN identified former patients from the Norwegian register of invasive cardiology. We included 16 patients whose MI event took place at least one year previously. All patients on the list were invited regardless of mental health status. The patients were contacted with a written invitation to participate. Those who wanted to participate replied directly to the project manager.

#### Measurements

All participants took part in an interview designed to probe into possible PTSD symptoms. All participants completed the Stressful Life Events Screening Questionnaire (SLESQ) and the PTSD checklist for DSM-5 (PCL-5), a validated clinical tool. Trained clinical psychologists conducted interviews and evaluations; however, this study did not include a complete diagnostic interview. We registered the number and types of PTEs with the SLESQ. The SLESQ scores are a continuous variable [32]. We assessed PTSD symptoms with the PTSD checklist (PCL-5). PCL-5 is a 20-item self-report measure that assesses the symptoms of PTSD according to DSM-5 [22]. It is used for screening individuals for PTSD and making a provisional PTSD diagnosis. Patients have the option to fill out the PCL-5 questionnaire before a session, while they are in a waiting room, or as part of a research study. The questionnaire typically takes 5–10 min to complete. Each item is formulated as a question such as "In the past month, to what extent were you bothered by: Repeated, disturbing, and unwanted memories of the stressful experience?" The response is measured on a 5-point Likert scale, ranging from 0 (not at all) to 4 (extremely). A PCL-5 score ≥ 31 is equivalent to probable PTSD. A lower cut-point score could be considered when screening or when it is desirable to maximize the detection of possible cases; however, we chose to use a moderate or higher cut-point score to make a probable diagnosis and to minimize false positives [24].

### Statistical analysis

In order to characterize the participants we observed, we examined their gender, the types of traumatic incidents they had encountered (PTEs), and their symptom severity levels based on the number of PTEs they had experienced. Based on our data analysis, we categorized the sample into three groups: "No PTE," "1–2 PTEs," and "3 or more PTEs". This differentiation was made due to the noticeable symptom differences observed at this threshold. We utilized Pearson's chi-square test to examine the associations among the amount of PTEs, gender, PTE categories, and the intensity of symptoms.

We performed a t-test to compare the frequency of PTEs in people with and without probable PTSD. We used ANOVA to investigate the relationship between the severity of symptoms and the prevalence of PTEs. The PCL-5 score was used as the dependent variable, while the number of PTEs was the independent variable. We used nonparametric series estimation with a polynomial basis to analyze potential thresholds. Nonparametric regression models the mean of the outcome based on the covariate without any assumptions about the functional relationship between the outcome and the covariates.

We determined the percentage of individuals who showed signs of probable PTSD during the clinical screening. To assess the prevalence of probable PTSD, we conducted a t-test comparing it to the hypothesized population prevalence of 2.5% [25, 26, 33].

Binary logistic regression analysis was conducted to model the association of PTEs with probable PTSD as response variable. We assessed the linearity assumption and checked for separation, collinearity, and outliers (no issues were found).

We used STATA v17 for all statistical analyses.

## Results

### Sample characteristics

All participants had experienced MI, but this did not necessarily imply they all found it traumatic: Seven participants reported no traumatic event, but most reported multiple PTEs (M = 2.6, SD = 2.2; Range 0 to 10). We observed that 60 (76.9%) participants reported MI as a traumatic event, but it was the primary traumatic event of only 22 (27.8%). Table 1 shows the sample characteristics stratified by the two sample groups. The main differences between the Tromsø7 and the hospital (UNN) samples was that the Tromsø7 participants were pre-screened on self-reported clinically significant symptoms in 2015/16, while the UNN sample was recruited from all patients. In the Tromsø7 sample, an average of 10.7 years had passed since the first MI, while for the UNN sample, their last MI was only one year previously. We observed that 11 (68.8%) participants reported MI as a traumatic event. We found no significant differences between the two samples except that the UNN sample reported more PTEs in the “Other” category.

**Table 1:** Sample characteristics for participants from the Tromsø Study and the University Hospital of North Norway

		TS sample		UNN sample		Full sample		*P*-value
**Categories of PTE (Obs/%)**^**a**^								
*Life-threatening disease*	No	13	21.0%	5	31.25%	18	23.08%	0.384
	Yes	49	79.0%	11	68.75%	60	76.92%	
*Physical violence*	No	36	58.1%	10	62.50%	46	58.97%	0.748
	Yes	26	41.9%	6	37.50%	32	41.03%	
*Psychological violence*	No	44	71.0%	11	68.75%	55	70.51%	0.862
	Yes	18	29.0%	5	31.25%	23	29.49%	
*Accidents*	No	37	59.7%	8	50.00%	45	57.69%	0.485
	Yes	25	40.3%	8	50.00%	33	42.31%	
*Sexual abuse or rape*	No	52	83.9%	14	87.50%	66	84.62%	0.720
	Yes	10	16.1%	2	12.50%	12	15.38%	
*Other*	No	50	84.7%	9	56.25%	59	78.67%	0.014
	Yes	9	15.3%	7	43.75%	16	21.33%	
**Potential traumatic events** **(SLESQ: Mean/SD)**^**b**^		2.65	(2.20)	2.94	(2.49)	2.71	(2.25)	0.633
**Symptom score** **(PCL-5: Mean/SD)**^**c**^		14.16	(13.89)	10.9	(9.99)	13.5	(13.2)	0.280

*Note*: *SD* Standard deviation, *PTE* Potential traumatic event(s), *SLESQ* Stressful Life Events Screening Questionnaire, *PCL-5* PTSD checklist for DSM-5, *TS* The Tromsø Study

^a^*P*-values are the result of Pearson chi-square for all cross-tabulations

^b^One-way ANOVA: F(1) = 0.23, *p* = 0.633

^c^One-way ANOVA: F(1) = 1.18, *p* = 0.280

### Categories of traumatic events and symptom severity

Table 2 shows the different categories of traumatic events and symptom severity (PCL-5). Despite the high prevalence of heart disease patients reporting an illness trauma, we found no direct association between illness trauma and symptom levels (*p* = 0.123). Physical violence (*p* = 0.002), psychological violence (*p* < 0.001), sexual abuse (*p* < 0.001), and other traumatic events (*p* = 0.042) were the categories that had an independent association with symptom levels.

**Table 2:** Potential traumatic event categories versus symptom severity. The Tromsø Study 2015–16

Category	Coefficient	95% confidence interval	F(1) value	*P*-value
Life-threatening illness	5.472	(-1.526, 12.471)	2.43	0.123
Physical violence	9.189	(3.473, 14.905)	10.25	0.002
Psychological violence	12.258	(6.316, 18.200)	16.88	< 0.001
Been in accident	5.879	(-0.033, 11.791)	3.92	0.051
Sexual abuse or rape	14.689	(7.096, 22.283)	14.85	< 0.001
Other traumatic events	7.452	(0.283, 14.621)	4.29	0.042

### Potential traumatic life events, symptom severity, and probable PTSD

We found nine participants (11.6%, 95% CI: 4.3% to 18.8%) with a PCL-5 cutoff score of 31 or above, indicative of *probable PTSD*. A one-sample t-test showed that the prevalence of 11.6% was significantly higher than the postulated population prevalence in Norway (Ha: mean > 0.025, t = 2.48, *p* < 0.015).

Exposure to PTE was found to be associated with the severity of symptoms and a probable PTSD. We conducted a one-way ANOVA analysis to examine the relationship between PTEs and symptom severity, as measured by PCL-5. Our findings revealed a significant linear trend, F(9) = 3.94, *p* < 0.001, omega sq. = 0.25, indicating that symptom severity increased proportionately as the number of PTEs increased. There were 76.9% male participants, but we found no sex differences in the number of reported PTEs (F(1) = 0.90, *p* = 0.346).

**Fig. 1:**
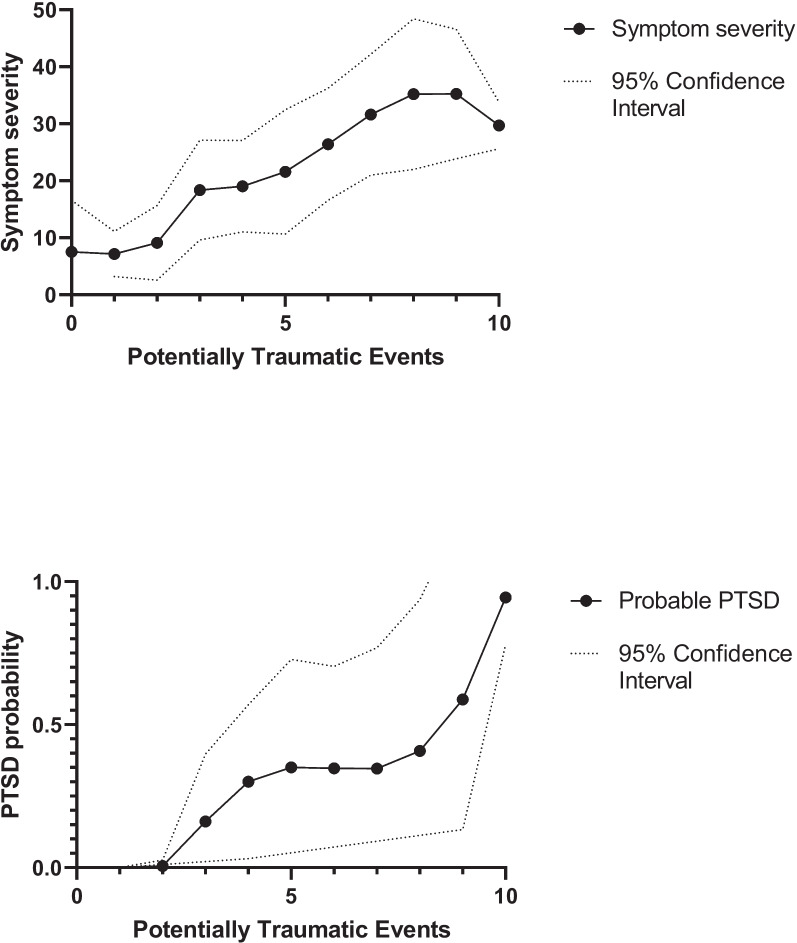
Spline model showing the association between the number of traumas and symptom severity in the Tromsø Study 2015–16

We estimated the odds ratio for probable PTSD with exposure to potentially traumatic events. The regression model revealed that the likelihood of probable PTSD increased by 76% (odds ratio 1.76, 95% CI: 1.26–2.47) for every one-unit increase in PTE (*N* = 78, Likelihood Ratio chi^2^ = 14.15, *p* < 0.001). Furthermore, as shown in Fig. 1, the splines suggest that three or more PTEs was the point at which the probability of developing PTSD significantly increased.

## Discussion

In this cross-sectional study of patients with myocardial infarction we studied the prevalence of potential traumatic events and PTSD, and found a clear correlation between the number of potentially traumatic events and the severity of symptoms. Nevertheless, we did not find a direct relationship between MI as an illness trauma and symptom levels. We identified that nine individuals were likely to have PTSD, which is significantly higher than the prevalence in the general population. However, after expanding our results to encompass the entire population of MI, we assessed the prevalence rate as 3.3% (Cf. supplementary file). Other studies have shown that the prevalence of PTSD in survivors of out-of-hospital cardiac arrest is around 12% (range 0%-38%), while the estimated occurrence of PTSD in this study is comparable to that of the general population in Norway [25, 26, 33].

There could be a number of reasons for this. Our analysis mainly centered on people exhibiting distinct symptoms, which means our estimation is conservative. The frequency of non-illness trauma should also be considered as a potential explanation for the prevalence rate. The participants were screened on average 10.6 years after their first MI, raising the possibility of remission during this period. This could be due to natural recovery, receiving correct treatment or support during that period. Other studies have observed that the prevalence of PTSD induced by MI tends to decrease within the first few years after the onset of the condition [34–38]. However, it is essential to note that some individuals may continue to experience post-traumatic symptoms for a prolonged period, despite the possibility of remission (Ibid.). One study revealed that about one-third of the participants still had PTSD up to two years after their MI episode [39], and a few studies indicate that symptoms worsened over time [40, 41].

Our findings indicate that while many participants experienced a traumatic event related to illness, only 35.5% of them identified MI as their primary trauma. Illness trauma was not directly related to PTSD symptom severity, whereas other forms of trauma and life-time exposure to PTE showed an independent association. This suggests that the mental health issues reported may be due to non-illness trauma. It is possible that other traumatic life events could lead to PTSD, which in turn increases the risk of cardiovascular disease. Additionally, research has shown that PTSD is a common psychological effect of heart disease events, which can worsen the patient’s prognosis. Therefore, it is important to regularly screen and monitor patients experiencing mental health issues.

## Clinical significance

Our study aimed to explore the intricate relationship between traumatic illness, the aftermath of a myocardial infarction (MI), and the development of post-traumatic stress disorder (PTSD). We acknowledge that this relationship can be complex, particularly when considering the role of non-illness traumas.

It is worth noting that there was no clear link between illness trauma and symptom levels, while other types of trauma did show a distinct association. This suggests that the mental health issues reported by this group may not solely be caused by illness trauma, but by other underlying factors. The data thus indicate that health personnel should inquire whether patients have experienced traumatic life events before their MI, rather than merely focusing on the MI event.

It is essential to screen and monitor patients with ongoing mental health issues. Early screening and targeted treatment can help prevent adverse effects on physical health outcomes [42]. Patients should, as a minimum, be informed about normal mental reactions due to traumatic life events and how they can care for their mental health after such experiences.

## Strengths and limitations

Sample characteristics are the primary threat to the external validity of the survey approach. The UNN sample was interviewed without prior knowledge of their mental health status and closer to the event than Tromsø7. However, the differences in sample characteristics were mostly non-significant. Upon separate analysis, we found a consistent association between PTEs, symptom severity, and probable PTSD.

The clinical assessment had high attendance rates, and the Tromsø Study is representative of the general population [30]. However, our sample from the Tromsø Study was limited, as we only included individuals with higher scores on the Hopkins Symptom Checklist-10. The number of eligible participants was therefore low and the high prevalence could be due to the sampling strategy, which included participants pre-screened for mental health symptoms. In Tromsø7, there were 818 MI patients, 208 of whom reported significant symptoms of anxiety or depression. Of the 140 patients still alive and invited, 62 participated in the study. Among them, we found an 11.6% prevalence of probable PTSD. However, based on this data, we cannot draw any conclusion on the population prevalence.

A ten-year gap may include new illnesses and other life events that are not reported, which could be a plausible reason why MI and illness trauma were unrelated. Further, the UNN sample was not pre-screened for mental health symptoms and was closer to the MI event. We found no significant differences between the two samples except that the UNN sample reported more PTEs in the “Other” category.

DSM-5 is the first manual to include life-threatening diseases such as PTEs that could lead to PTSD, while ICD-10 uses more general terms such as “exceptionally threatening or catastrophic situation”. Using DSM-5 may make our results less comparable to previous studies using other diagnostic tools, but it allowed us to investigate MI specifically as a PTE.

The cross-sectional design was a limitation as we did not examine clinical change relative to the MI event. Future research should include a structured diagnostic interview, including all eligible patients, and treatment outcome research to address these limitations and develop the initial findings.

The statistical conclusion validity is limited. The estimated statistical power of an association of illness trauma and symptom levels was 0.137 at an alpha of 5%. It is therefore impossible to conclude that there was no connection. The estimated power of the number of PTEs and symptom levels was excellent (0.997).

## Conclusion

A likely PTSD diagnosis correlated with the number of potentially traumatic events but not directly with previous experience of a life-threatening disease. There was no evidence that PTSD is a typical comorbid condition of MI, although PTSD is likely in patients who report mental distress after MI. Further research is needed to explain why PTSD occurs more frequently among heart disease patients.

### Supplementary Information

**Additional file 1.**

## Data Availability

Data from the Tromsø Study is available for researchers who meet the criteria for access to confidential data (https://en.uit.no/prosjekter/prosjekt?p_document_id=80172). Readers may also contact Professor Sameline Grimsgaard, sameline.grimsgaard@uit.no, to request the data or receive confirmation that data will be available to all interested researchers upon request. http://tromsoundersokelsen.uit.no/webview/

## Abbreviations

CVD: Cardiovascular disease
HSCL-10: Hopkins Symptom Checklist score
MI: Myocardial infarction
PCL-5: The PTSD checklist for DSM-5
PTE: Potential Traumatic Event
PTSD: Post-traumatic stress disorder
SLESQ: Stressful Life Events Screening Questionnaire
UNN: University Hospital of North Norway
