# Supplementary material for: Post-traumatic stress disorder among heart disease patients: a clinical follow-up of individuals with myocardial infarction in the Tromsø Study
Source: BMC Psychiatry. 2023 Dec 12;23:936. doi: 10.1186/s12888-023-05431-2 (PMC10714632; doi:10.1186/s12888-023-05431-2)
Supplement: Supplementary file 1 — Additional file 1. [file 12888_2023_5431_MOESM1_ESM.docx]

The estimated prevalence of probable PTSD in the Tromsø7 MI population.

We observed that 11.6% (95% CI: 4.3% to 18.8%) of the 78 participants had a PCL-5 cutoff score of 31 or above, indicating probable PTSD. The lifetime prevalence of PTSD is 2.5% in Norway and approximately 8% in the USA (22-24). We wanted to compare the observed probable PTSD prevalence against a hypothesized population prevalence.

We employed Multiple Imputations to evaluate the overall prevalence of PTSD in the MI population. In Tromsø7, the total number of MI patients was 818, out of which 208 had reported significant symptoms. We assumed that individuals without mental health symptoms were unlikely to have PTSD. We also assumed that the prevalence of PTSD in our sample (11.6%) was representative of all those with significant symptoms of anxiety or depression. We made 100 imputations by logistic regression, using age and sex as independent variables. The estimated prevalence of PTSD among the MI population in the Tromsø Study was 3.3% (95% CI: 1.3% to 5.2%), which was not significantly different from the assumed population prevalence of 2.5%.
